# Detection of Gag C-terminal mutations among HIV-1 non-B subtypes in a subset of Cameroonian patients

**DOI:** 10.1038/s41598-022-05375-9

**Published:** 2022-01-26

**Authors:** Georges Teto, Alex Durand Nka, Joseph Fokam, Yagai Bouba, Désiré Takou, Lavinia Fabeni, Luca Carioti, Daniele Armenia, Ezéchiel Ngoufack Jagni Semengue, Béatrice Dambaya, Samuel Martin Sosso, Vittorio Colizzi, Carlo-Federico Perno, Francesca Ceccherini-Silberstein, Maria Mercedes Santoro, Alexis Ndjolo

**Affiliations:** 1 Chantal BIYA International Reference Centre for Research on HIV/AIDS Prevention and Management (CIRCB), Yaoundé, Cameroon; 2 University of Rome “Tor Vergata”, Rome, Italy; 3 Evangelical University of Cameroon, Bandjoun, Cameroon; 4 Faculty of Health Science, University of Buea, Buea, Cameroon; 5 National HIV Drug Resistance Working Group, Ministry of Public Health, Yaoundé, Cameroon; 6 Laboratory of Virology, National Institute for Infectious Diseases “Lazzaro Spallanzani”-IRCCS, Rome, Italy; 7 Saint Camillus International, University of Health and Medical Sciences, Rome, Italy; 8 Bambino Gesu Pediatric Hospital, Rome, Italy

**Keywords:** Virology, Antivirals, Retrovirus, Antimicrobial resistance

## Abstract

Response to ritonavir-boosted-protease inhibitors (PI/r)-based regimen is associated with some Gag mutations among HIV-1 B-clade. There is limited data on Gag mutations and their covariation with mutations in protease among HIV-1 non-B-clades at PI/r-based treatment failure. Thus, we characterized Gag mutations present in isolates from HIV-1 infected individuals treated with a PI/r-regimen (n = 143) and compared them with those obtained from individuals not treated with PI/r (ART-naïve [n = 101] or reverse transcriptase inhibitors (RTI) treated [n = 118]). The most frequent HIV-1 subtypes were CRF02_AG (54.69%), A (13.53%), D (6.35%) and G (4.69%). Eighteen Gag mutations showed a significantly higher prevalence in PI/r-treated isolates compared to ART-naïve (p < 0.05): Group 1 (prevalence < 1% in drug-naïve): L449F, D480N, L483Q, Y484P, T487V; group 2 (prevalence 1–5% in drug-naïve): S462L, I479G, I479K, D480E; group 3 (prevalence ≥ 5% in drug-naïve): P453L, E460A, R464G, S465F, V467E, Q474P, I479R, E482G, T487A. Five Gag mutations (L449F, P453L, D480E, S465F, Y484P) positively correlated (Phi ≥ 0.2, p < 0.05) with protease-resistance mutations. At PI/r-failure, no significant difference was observed between patients with and without these associated Gag mutations in term of viremia or CD4 count. This analysis suggests that some Gag mutations show an increased frequency in patients failing PIs among HIV-1 non-B clades.

## Introduction

For several decades, Human Immunodeficiency Virus (HIV) has caused many deaths. In fact, 150,000 [100,000–210,000] AIDS related deaths were recorded in Western and Central Africa^1^, making this region the most affected by the pandemic, despite the scale-up of antiretroviral therapy (ART). In the current era of "Test and Treat" in resource-limited settings (RLS), there is a need to closely monitor and study determinants of poor therapeutic outcomes, so as to ensure a sustained long-term efficacy of available drug regimens^2^. Such goals are of paramount importance; as viral suppression remains suboptimal (only 81.1% of adults on ART, below the expected 95% target for achieving the elimination goal by 2030)^2^. Some of the reasons for the poor viral suppression rate in people on treatment include non-adherence to treatment^3^, interruptions of treatment^4^, and very-high baseline viremia^5^, which generally contribute substantially to the emergence of resistant viral strains^6^. With long-term exposure to ART and switch of treatment, the number of HIV-infected patients receiving ritonavir boosted protease inhibitors (PI/r)-based ART is increasing substantially in RLS. Of note, PI/r represents the backbone of first-line ART in children, and is the core-molecule for second-line ART in adolescents/adults, as recommended by the World Health Organization for RLS, with essentially regimens containing Lopinavir/ritonavir (LPV/r) or ritonavir-boosted Atazanavir (ATV/r)^7^. Viral resistance to PI/r evolves initially by mutations in the protease region. Mutated residues in HIV protease are classified as either major or minor resistance mutations, according to their effect on ART clinical outcomes^8^. Following the Stanford algorithm (mutation list), minor resistance mutations (L10F, V11I, K20TV, L23I, L33F, K43T, F53L, Q58E, A71IL, G73STCA, T74P, N83D, and L89V)^9^ are assumed to have ancillary roles such as compensation for lower efficiency of proteolysis caused by major mutations; major resistance mutations (V32I, M46IL, I47VA, G48VM, I50VL, I54VTALM,L76V,V82ATFS, I84V,N88S, L90M)^9^ tend to confer high levels of resistance to one or multiple PI/r and develop early in patient treatment^10^.

Specifically, the emergence of HIV resistance to PI/r requires a stepwise accumulation of primary and compensatory mutations in the viral protease. Additionally, selected Gag (Group of specific antigens) mutations have been recently shown to provide compensatory functions for PR resistance mutations, which may contribute to poor treatment outcomes on PI/r-containing regimens due to the emergence of Gag-specific drug selected mutations among B subtype^11,12^. As mechanism of resistance may differ between B and non-B mutations, studies on non-B mutations are limited.

The HIV protease is essential for cleaving the Gag and Gag-pol polyproteins into their functional protein products, leading to the assembly of a mature infectious virus particle^13–15^. The protease has five cleavage sites on the Gag gene: the first cleavage separates the nucleocapsid protein (NC) from the capsid protein (CA) downstream of the 14 amino acid binding peptide called spacer peptide 1 (SP1); the capsid is subsequently separated from the matrix protein (MA), which remains associated with the virion membrane^13–15^; this event is almost simultaneous to the release of the C-terminal p6 Gag protein, downstream of another linker peptide located between NC and p6, termed SP2 (spacer peptide 2, formerly termed p1); finally, the two linker peptides SP1 and SP2 are trimmed from the CA and NC proteins, respectively^13–15^. Of note, mutations in Gag ere reported to be contribute substantially to PI/r resistance besides compensating for fitness loss^11,12^. These potential effects are essentially driven by the C-terminal region, with little contribution from MA, CA and SP1^11^.

Some adherent patients failing treatment on PI/r-based ART do not harbor any major protease mutations, thus suggesting the detrimental effects of closer genes like Gag on the resulting poor treatment response^16^. This calls for further investigation on Gag genes for a successful scaling-up of PI/r-based ART in RLS like Cameroon. Of note, the role of drug resistance mutations in HIV protease has been studied extensively, whereas mutations in its substrate Gag have not been thoroughly ascertained.

A better understanding of HIV-1 Gag gene mutations and their co-variation with protease mutations among patients failing on PI/r-based regimen might be of great clinical relevance, especially as failure under PI without resistance is common.

We therefore sought to determine P7 (NC)-P6 HIV-1 Gag gene mutations selected under PI/r pressure and their covariations with protease mutations among HIV-1 non-B clades.

## Results

### Demographic and clinical characteristics of study participants

Our study population consisted of 362 PLHIV, of whom 101 were ART-naïve, 118 on a regimen containing only RTIs and 143 on a regimen containing a PI/r. Females were predominant (57.2%, 207/362), and the median [IQR] age was 41 [33–49] years. ART-naïve patients showed a significantly lower age compared to those treated (median [IQR], years: 38 [31–48] vs. 44 [32–52] in NNRTI-treated patients vs. 41 [33–47] in PI/r-treated patients; p = 0.031). For those on treatment, the median duration on ART was 63 [IQR: 35–105] months. The overall median [IQR] CD4 cells count was 181 [60–361] cell/mm3, with no statistically significant difference observed among the three groups. Regarding viremia, ART-naïve patients had the highest median [IQR] viral load (5.6 [5.4–6.7] log_10_ copies/mL), followed by those on RTI-based regimens (5.3 [4.7–5.7] log_10_ copies/mL; versus on PI/r-based regimens (5.1 [4.3–5.6] log10 copies/mL; p < 0.001). According to ART regimen, the majority of patients on RTI received TDF + 3TC + EFV (52.5%), followed by AZT + 3TC + NVP (19.5%); of the patients on PI/r, the majority received LPV/r or ATV/r containing regimen (65.7% and 32.9%, respectively) (Table 1).

**Table 1:** Socio-demographic characteristics of the study population.

	Naïve (n = 101)	RTI-treated (n = 118)	PI/r-treated (n = 143)	p-value^a^
**Gender**				
Female n (%)	59 (28.5)	73 (35.3)	75 (36.2)	0.516
Male n (%)	42 (27.1)	45 (29.0)	68 (43.9)	
Age (years), median [IQR]	38 [31–48]	44 [35–52]	41 [33–47]	0.031
CD4 (cells/μl), median [IQR]	231 [60–340]	169 [58–393]	167 [61–362]	0.760
Viral load (Log_10_/copies/mL), median [IQR]	5.61 [5.4–6.7]	5.3 [4.7–5.7]	5.1 [4.3–5.6]	< 0.0001
**ART regimen n (%)**				
2NRTIs + EFV	–	69 (58.4)	–	
2NRTIs + NVP	–	49 (41.5)	–	
2 NRTIs + LPV/r	–	–	94 (65.7)	
2 NRTIs + ATV/r	–	–	47 (32.9)	
2 NRTIs + IDV/r	–	–	2 (1.4)	

IQR: Interquartile range; NRTIs: Nucleoside reverse transcriptase inhibitors; EFV: Efavirenz; NVP: Nevirapine; LPV/r: Lopinavir; ATV/r: Atazanavir; IDV/r: Indinavir; PI/r: ritonavir boosted protease inhibitors; RTI: Reverse transcriptase inhibitors.

^a^Chi-squared of independence test was used to estimate the potential differences.

### Viral subtypes distribution and protease drug resistance mutations profile among PI/r treated patients

**Figure 1:**
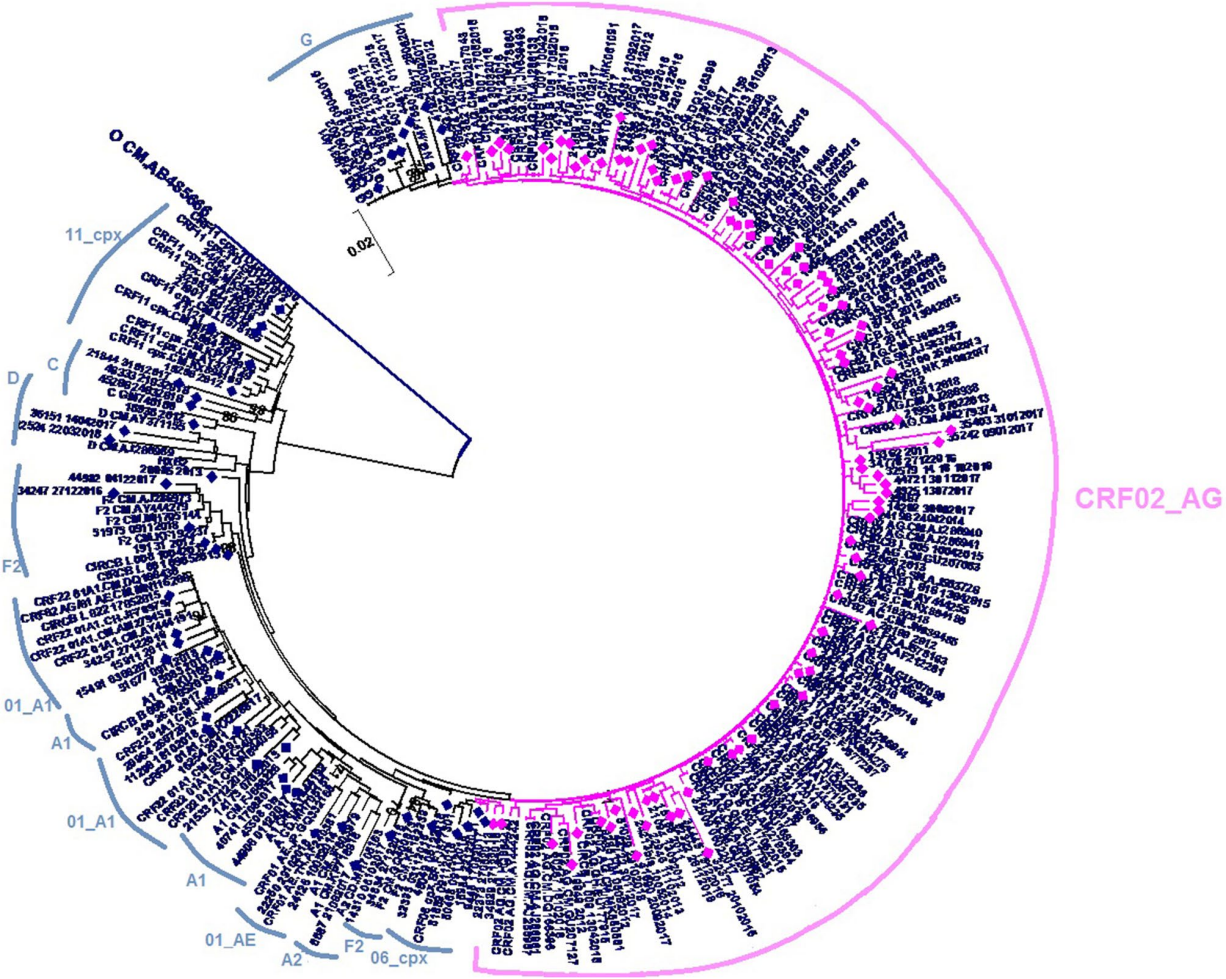
Phylogenetic tree of the 143Viral subtypes distribution among HIV-1 infected patients failing PI/r treatment, inferred using MEGA version 5.0 software (https://www.megasoftware.net/); the scale bar represents 2% genetic distance.

Out of the overall 362 study participants, a broad diversity of HIV-1 non-B clades was found, driven by CRF02_AG (198, 54.69%), followed by pure subtypes A (49, 13.53%), D (23, 6.35%) and G (17, 4.69%). Among the 143 PI/r treated patients, the most represented subtype was also CRF02_AG subtype (87, 60.83%), follow by subtype A1 (22, 13.98%); G (12, 8.39%); CRF_11.cpx (9, 6.29%); F2 (6, 4.19%); D (2, 1.39%); C (2, 1.39%); CRF06_cpx (1, 0.69%), A2 (1, 0.69%); CRF01_AE (1, 0.69%) (Fig. 1).

**Figure 2:**
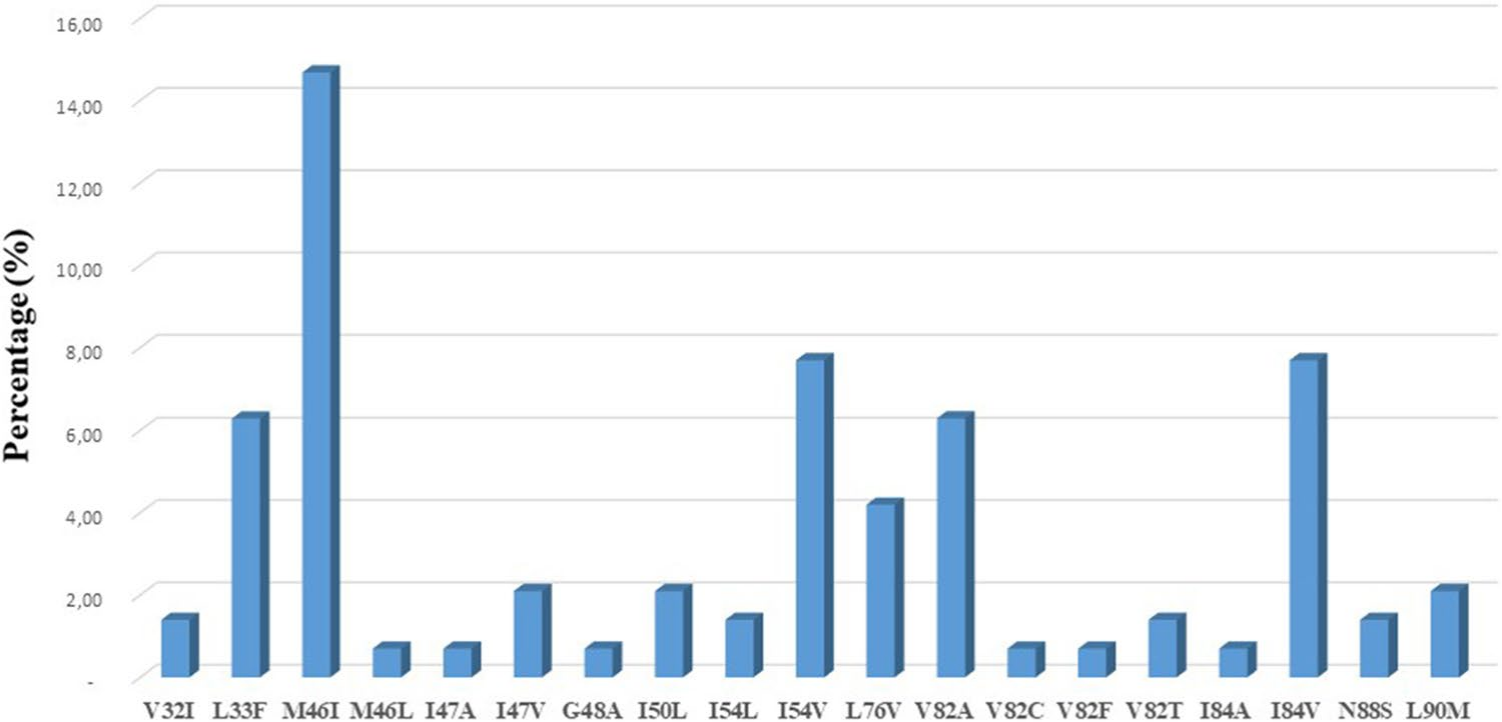
Distribution of major protease drug resistance mutation. Resistance mutations were defined according to the Stanford algorithm.

The overall prevalence of the presence of at least one protease drug resistance mutation among PI/r-experienced patients was 19.5% (n = 28). The most frequent mutations were M46I (21; 14.69%), I84V (11, 7.69%) and I54V (11, 7.69%) (Fig. 2).

### P7 (NC)-P6 Gag mutations associated to PI/r exposure

By evaluating the last 56 amino acids of Gag sequences derived from 101 drug-naïve and 143 patients on PI/r containing regimen, we identified 18 mutations associated to PI/r exposure, based on the assumption that these mutations occurred with different frequencies in ART-naïve patients compared to patients on PI/r-based regimen.

**Figure 3:**
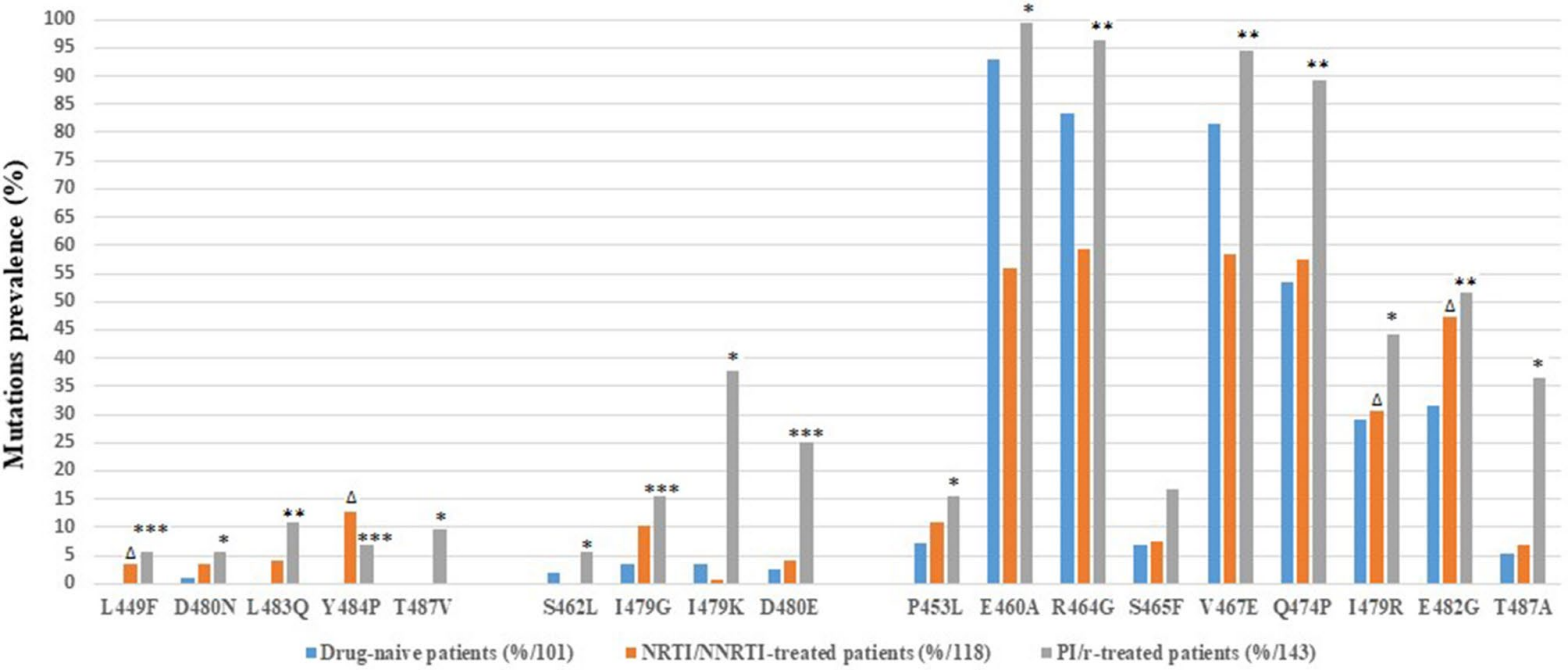
HIV-1 Gag gene mutations with significant increase in frequency from isolates between drug-naive and NRTI/NNRTI and/or PI/r treated patients. Statistical differences were assessed by chi-squared tests of independence (PI/r vs. ART-naïve and RTI vs. ART-naïve). p-values were significant at a false discovery rate of 0.05 following correction for multiple comparison. *p < 0.05; **p < 0.001; ***p < 0.0001 for PI/r treated patients vs. those ART naïve. Δ p < 0.05 for the comparison between NRTI/NNRTI vs. ART naïve. NNRTI: non-NRTI, NRTI: nucleoside reverse transcriptase inhibitor, PI/r: ritonavir boosted protease inhibitors, RTI: reverse transcriptase inhibitor.

These mutations were grouped into three classes, based on their prevalence in isolates from treatment naïve and PI/r treated individuals (Fig. 3).

Class I included five mutations (L449F, D480N, L483Q, Y484P, T487V) that were completely absent or occurred with a frequency of < 1% in isolates from drug-naïve patients and showed a significant increase in isolates from patients on PI/r-based regimen (5.59–11.18%).

Class II included four mutations (S462L, I479G, I479K, D480E) already present in isolates from drug naïve patients at a frequency between 1 and 5% but with a significant increase in isolates from patients on PI/r-based regimen (5.59–37.76%).

Class III included nine mutations (P453L, E460A, R464G, S465F, V467E, Q474P, I479R, E482G, T487A) already present in isolates from drug naïve patients at a frequency ≥ 5% but with a significant increase in isolates from PI/r-based patients (15.38–99.31%).

### Gag P7(NC)-P6 mutations significantly associated to both reverse transcriptase inhibitors (RTI) and PI/r exposure

Four Gag P7(NC)-P6 mutations significantly associated to RTI exposure compared to naïve patients were also significantly associated to PI/r exposure (L449F, p = 0.032; I479R, p = 0.002; E482G, p = 0.012; Y484P, p < 0.0001) (Fig. 3). Other Gag mutations P453L, E460A, S462L, R464G, S465F, V467E, Q474P, I479G, I479K, D480E, D480N, L483Q, T487A and T487V were only associated with PI/r exposure and not RTI when compared to ART naïve patients. Among the 18 Gag mutations significantly associated to PI/r exposure, ten mutations (P453L; E460A; R464G; V467E; I479K; Q474P; I479K; D480E; T487A; T487V) significantly differed between isolates from PI/r-treated vs RTI-treated (p < 0.05, Fig. 3).

### Gag P7(NC)-P6 mutations according to HIV-1 viral subtypes

Of the 18 mutations significantly associated to PI/r exposure, seven mutations were statistically different among HIV-1 subtypes. Indeed, in class 1, mutations L483Q, Y484P and T487V showed a significantly higher prevalence among subtype A1 infected individuals, when compared to other subtypes (p < 0.001, Table 2). Of note, all the mutations with significantly varying frequencies of class 1 were found only in subtype A1 and other (mainly made of CRF11_cpx which has a portion of A subtype in Gag region). Regarding class 2, I479K was significantly more frequent in subtype categorized as other and D480E in subtype G, p < 0.001. for class 3, S465F was significantly more frequent in subtype categorized as other and E482G in CRF02_AG, p < 0.001.

**Table 2:** Gag P7(NC)-P6 mutations according to HIV-1 viral subtypes.

Class	Mutations	Overall n (%)	Subtypes n (%)				
			CRF02_AG (n = 87)	A1 (n = 22)	G (n = 12)	Others (n = 22)	p-value^b^
I	L449F	8 (5.59)	3 (3.44)	1 (4.54)	1 (8.33)	3 (13.63)	0.305
	D480N	8 (5.59)	3 (3.44)	2 (9.09)	0 (0.00)	3 (13.63)	0.450
	L483Q	16 (11.18)	0 (0.00)	11 (50.00)	0 (0.00)	5 (22.72)	< 0.001
	Y484P	10 (6.99)	0 (0.00)	6 (27.27)	0 (0.00)	4 (18.18)	< 0.001
	T487V	14 (9.79)	0 (0.00)	11 (50.00)	0 (0.00)	3 (13.63)	< 0.001
II	S462L	8 (5.59)	4 (4.59)	3 (13.63)	0 (0.00)	1 (4.54)	0.273
	I479G	22 (15.38)	18 (20.68)	3 (13.63)	1 (8.33)	0(0.00)	0.161
	I479K	54 (37.76)	25 (28.73)	7 (31.81)	4 (33.33)	18(81.81)	< 0.001
	D480E	36 (25.17)	16 (18.39)	1 (4.54)	9 (75.00)	10(45.45)	< 0.001
III	P453L	22 (15.38)	10 (11.49)	6 (27.27)	5 (41.66)	1 (4.54)	0.190
	E460A	142 (99.30)	86 (98.85)	22 (100.00)	12 (100.00)	22 (100.00)	1.000
	R464G	138 (96.50)	87 (100.00)	18 (81.81)	11 (91.66)	22 (100.00)	0.403
	S465F	24 (16.78)	0 (0.00)	0 (0.00)	8 (66.66)	16 (72.72)	< 0.001
	V467E	135 (94.40)	87 (94.25)	17 (77.27)	12 (66.66)	19 (86.36)	0.576
	Q474P	99 (69.23)	57 (65.51)	16 (72.72)	4 (33.33)	22 (100.00)	0.320
	I479R	63 (44.05)	39 (44.82)	10 (45.45)	5 (41.66)	9 (40.90)	0.162
	E482G	74 (51.74)	68 (78.16)	2 (9.09)	2 (16.66)	2 (9.09)	< 0.001
	T487A	52 (36.36)	36 (41.37)	3 (13.63)	5 (41.66)	8 (36.36)	0.139

^a^Eighteen (18) Gag mutations significantly associated to PI/r exposure.

^b^Chi-squared of independence test was used to estimate the overall potential differences among types. The subtypes categorized as “other” included: CRF11_cpx (9), F2 (6), D (2), C (2), CRF06_cpx (1), A2 (1) and CRF01_AE (1).

### Covariation of Gag mutations with protease mutations

Another goal of our study was to assess the covariation of HIV Gag P7(NC)-P6 mutations with other mutations observed in the protease gene of 143 PI/r-treated patients, focusing on PI/r major and/or accessory drug resistance mutations according to the Stanford list of mutations^9^.

To identify significant patterns of pairwise correlations between Gag P7 (NC)-P6 mutations and protease mutations observed in isolates from PI/r-treated patients, we calculated the binomial correlation coefficient (phi) and its statistical significance for each pair of mutations (Table 3).

**Table 3:** Significantly correlated pairs of HIV-1 Gag mutations with protease major or accessory resistance mutations.

Class	Gag mutations	Frequency (%)^a^	Covariated mutations	Frequency (%)^b^	Covariation frequency (%)	phi	p-value^d^
I	L449F	8 (5.59)	L10F	6 (4.19)	2 (25.00)	0.25	3.69E−02
			**V32I**	3 (2.09)	2 (25.00)	0.38	8.04E−03
			**M46I**	22 (15.38)	4 (50.00)	− 0.07	1.98E−02
			**I54V**	11 (7.69)	3 (37.5)	0.27	1.55E−02
			**L90M**	4 (2.79)	2 (25.00)	0.32	1.56E−02
	Y484P	10 (6.99)	K20T	4 (2.79)	2 (20.00)	0.28	2.46E−02
			**L33F**	9 (6.29)	3 (30.00)	0.26	1.68E−02
			**I54V**	11 (7.69)	4 (40.00)	0.33	3.23E−03
			**V82A**	9 (6.29)	3 (30.00)	0.26	1.68E−02
			L89V	8 (5.59)	3 (30.00)	0.29	1.16E−02
II	I479G	22 (15.38)	**I64M**	19 (13.28)	7 (31.81)	− 0.09	1.18E−02
	D480E	36 (25.17)	**V82A**	9 (6.29)	5 (13.88)	0.18	4.47E−02
III	P453L	22 (15.38)	L10F	6 (4.19)	3 (13.63)	0.20	4.68E−02
			**L33F**	9 (6.29)	5 (22.72)	0.28	4.62E−03
			**M46I**	22 (15.38)	9 (40.90)	0.30	1.35E−03
			**I54L**	2 (1.39)	2 (9.10)	0.27	2.28E−02
			**I54V**	11 (7.69)	7 (31.81)	0.38	1.79E−04
			Q58E	6 (4.19)	3 (13.63)	0.20	4.68E−02
			**V82A**	9 (6.29)	4 (18.18)	0.20	3.19E−02
			**V82T**	2 (1.39)	2 (9.10)	0.27	2.28E−02
			**I84V**	11 (7.69)	7 (31.81)	0.38	1.79E−04
			L89V	8 (5.59)	5 (22.72)	0.31	2.29E−03
	S465F	24 (16.78)	**I54V**	11 (7.69)	5 (20.83)	0.22	2.03E−02
			**V82A**	9 (6.29)	4 (16.67)	0.19	4.35E−02
	Q474P	99 (69.23)	**M46I**	22 (15.38)	11 (11.11)	− 0.03	4.46E−02
	E482G	74 (51.74)	**I54V**	11 (7.69)	2 (2.70)	− 0.19	2.71E−02
	T487A	52 (36.36)	**M46I**	22 (15.38)	3 (5.76)	− 0.21	1.64E−02

^a^The frequency was determined in 143 isolates from PI/r-treated patients.

^b^Percentages were calculated for patients containing each specific mutation.

^c^All P values for covariation were significant at a false discovery rate of 0.05. Mutations in bold represents major protease resistance mutations.

#### Gag P7(NC)-P6 mutations involved in positive correlations with PI/r major resistance mutations

In Class I, two mutations were positively correlated (phi > 0, p  < 0.001) as pairs with protease major resistance mutations: L449F correlated with the major resistance mutations V32I (phi = 0.38, p = 0.008), I54V (phi = 0.27, p = 0.015) and L90M (phi = 0.32, p = 0.015); Y484P correlated with the major mutations I54V (phi = 0.33, p = 0.003) and V82A (phi = 0.26, p = 0.016). In Class II, D480E positively correlated with the major mutations I54V (phi = 0.31, p =  p < 0.001) and V82A (phi = 0.26, p = 0.044). In Class III, only the Gag mutation P453L positively correlated with some major mutations as follows: L33F (phi = 0.28, p = 0.004), M46I (phi = 0.30, p = 0.001), I54L (phi = 0.27, p = 0.022), I54V (phi = 0.38, p < 0.001), V82A (phi = 0.20, p = 0.031), V82T (phi = 0.27, p = 0.021), and I84V (phi = 0.38, p < 0.001), (Table 3).

#### Gag P7(NC)-P6 mutations involved in negative correlations with PI/r major resistance mutations

In Class I Gag mutations, L449F showed a significant negative correlation (phi < 0 and p < 0.05) with M46I (phi = − 0.07, p = 0.019); in Class II, I479G correlated with I64M (phi = − 0.09, p = 0.011); in class III, E482G correlated with I54V (phi = − 0.19, p = 0.027), Q474P with M46I (phi = − 0.03, p = 0.044) and T487A with M46I (phi = − 0.21, p = 0.016) (Table 3).

#### Gag P7(NC)-P6 mutations involved in correlations with PI/r non polymorphic accessory resistance mutations

Three Gag C-terminal mutations significantly associated with PI/r exposure showed significant positive correlations with specific PI/r non polymorphic accessory resistance mutations. In particular L449F correlated with L10F (phi = 0.25, p = 0.036); P453L with L10F (phi = 0.20, p = 0.046), Q58E (phi = 0.20, p = 0.046) and L89V (phi = 0.31, p = 0.002); Y484P with three PI/r accessory resistance mutations K20T (phi = 0.28, p = 0.024), and L89V (phi = 0.29, p = 0.011) (Table 3).

### Clusters of correlated mutations

Because pairwise analysis suggested that Gag mutations are associated with specific evolutionary pathways of known resistance-conferring mutations, we performed average linkage hierarchical agglomerative cluster analysis^17^ to investigate this hypothesis in more detail.

**Figure 4:**
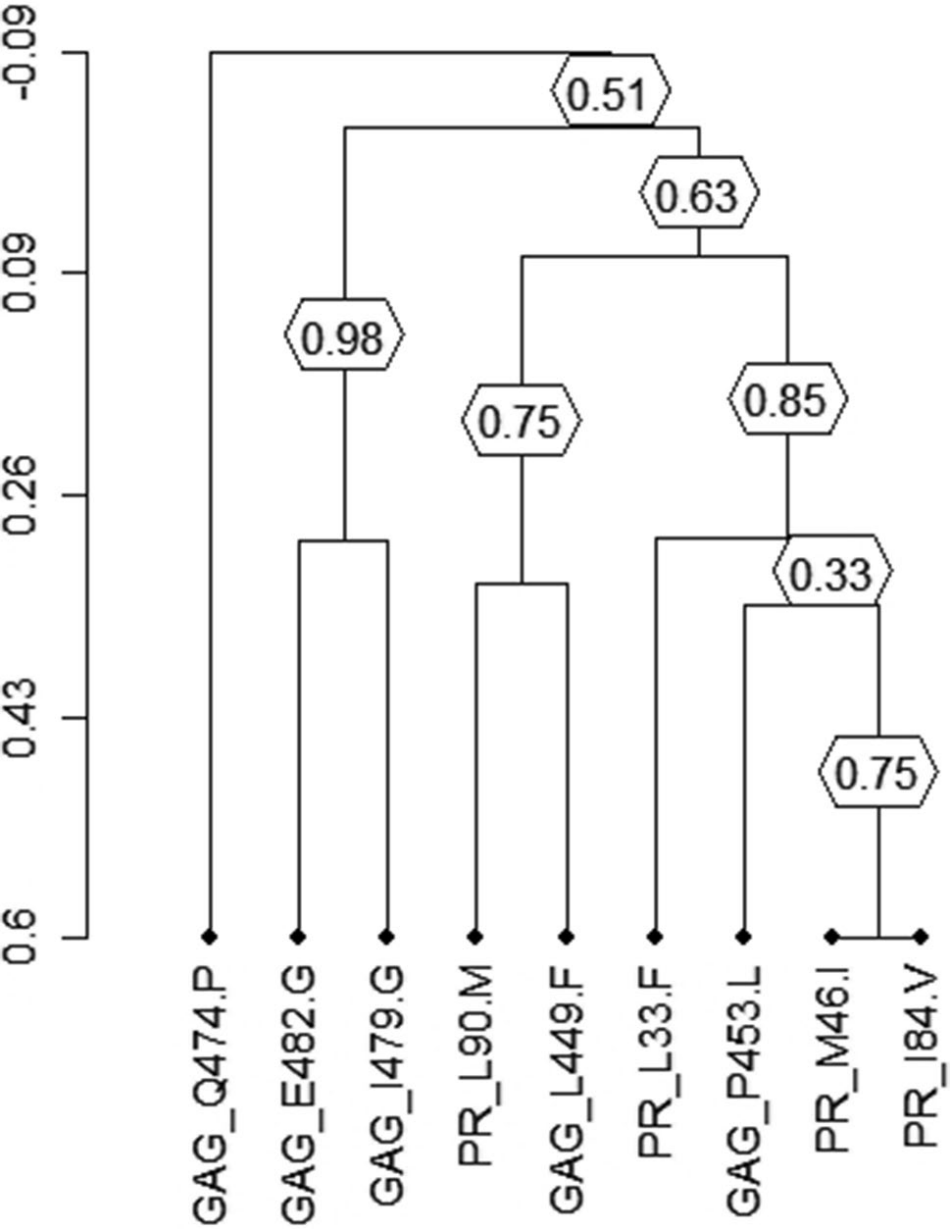
Clusters of correlated mutations. Dendrogram obtained from average linkage hierarchical agglomerative clustering, showing clusters of Gag mutations and protease resistance mutations. The length of branches reflects distances between mutations in the original distance matrix. Bootstrap values, indicating the significance of clusters (≥ 0.2), are reported in the boxes. Gag mutations start with ‘Gag’ and protease mutations with ‘PR’.

The dendrogram (Fig. 4) shows that Gag mutation L449F and P453L significantly correlated to PI/r major resistance mutations. Specifically, P453L clustered (bootstrap value = 0.33) with major PI/r resistance mutations M46I and I84V (covariation frequency: 40.9% and 31.8%, respectively). Likewise, another cluster was formed by L449F and L90M (bootstrap value = 0.75, covariation frequency: 25.0%).

### Association of Gag P7(NC)-P6 mutations positively correlated to PI/r major resistance mutation with viral load and CD4 cell count at failure.

A further step in our study was to assess the difference in HIV viral load and CD4 cell count at the time of genotypic test between the patients harboring the Gag P7(NC)-P6 mutations positively correlated to PI/r major resistance mutations and those with wild type amino acids. Despite the slightly high median viremia in presence of two Gag mutations (L449F and Y484P) when compared to individuals with a wild-type residues, no significant variation was found in terms of viremia. Similarly, even though the median CD4 cells count was lower in all patients with Gag mutations when compared to wild type residues, no significant difference was found between the two groups in term of CD4 cell count (Table 4).

**Table 4:** Comparison of viremia and CD4 count of patients with wild type and mutated (positive correlated) amino acid.

Position	Residue	Frequency n (%) ^a^	Viremia, median [IQR] copies/ml^b^	Ratio	p-value^c^	CD4 count, median [IQR] cells/mm^3b^	Ratio	p-value^c^
449	L^d^	18 (12.68)	162,116 [3725–186,450]	3.31	0.468	166 [46–443]	0.71	0.653
	F	8 (5.59)	537,857 [2689–720,471]			118 [35–322]		
484	Y^d^	23 (16.08)	90,407 [4172–119,019]	2.46	0.388	126 [74–467]	0.60	0.851
	P	10 (6.99)	222,565 [6093–298,322]			107 [32–306]		
480	D^d^	88 (61.53)	255,855 [3747–318872]	0.20	0.184	178 [78–489]	0.69	0.736
	E	36 (25.17)	51,807 [2226–74,908]			124 [69–436]		
453	P^d^	116 (81.11)	172,291 [6785–245,815]	0.50	0.353	127 [81–351]	0.73	0.078
	L	22 (15.38)	87,814 [5535–98,,671]			93 [31–317]		

^a^The frequency was determined in 143 PI-treated patients.

^b^Viremia and CD4 cell count values are contextual (± 30 days) to the genotype resistance testing and were available for 110 PI/r treated patients (77% of samples).

^c^p-values were determined by Mann–Whitney test.

^d^The wild-type amino acid in consensus B.

## Discussion

In this study we identified eighteen HIV-1 Gag mutations which are significantly associated with exposure to PIs. These findings suggest that HIV-1 Gag P7(NC)-P6 mutations was associated to PI/r regimen among HIV-1 non-B subtypes.

Some HIV-1 Gag P7 (NC)-P6 mutations (L449F, D480N, L483Q, Y484P, T487V) which were rare or completely absent in isolates from ART-naïve patients had a significantly increased frequency among PI/r treated isolates at virological failure. This suggests that these mutations might be selected under PI/r pressure. These mutations have also been documented by previous studies as being associated with PI/r regimen^11,16,18^. Moreover, class I mutations (e.g., L449F and Y484P) occurred principally in combination with several major PI/r resistance mutations, suggesting that they emerge after a prolonged PI/r exposure, when the virus has already accumulated a large number of PI/r resistance mutations. In this regard, a previous study demonstrated that the emergence of protease major resistance mutation I50V require as a prerequisite changes in the Gag gene at position L449 in vivo and cause reduction of sensitivity to amprenavir and an improved viral fitness in vitro^18^*.* The same observation was made in another study where this mutation was present exclusively among individuals failing PI/r-based treatment^19^. Protease mutation L90M had the strongest correlation with L449F which is confirmed in the dendrogram. These major protease mutations I50V and L90M would therefore be a sentinel for the L449F in the Gag gene. Gag Y484P mutation was shown to be associated to darunavir exposure and was classified as a novel Gag C-terminus mutation associated to PI/r regimen^20^.

We have also observed that some mutations (class II: S462L, I479G, I479K and D480E) which were already moderately present (1–5%) in isolates from ART-naïve patients significantly increased their prevalence (positive association) in isolates from patients on PI/r regimen. Mutations S462L and I479K were previously identified as Gag polymorphisms which are associated to PI/r exposure^21^. Also, Gag mutation D480E which is recognized as a PI-exposure associated mutation^21^ significantly correlated with two major PI/r resistance mutations (I54V and V82A). In the other hand, given that some studies have shown that only Gag mutations are capable of inducing resistance to darunavir^16^, the role of the novel mutation I479G which did not significantly correlate with any major or accessory PI/r resistance mutation deserve to be investigated.

Among the mutations already present in isolates from ART-naïve patients at a frequency of ≥ 5% but with a significant increase in isolates from PI/r treated patients (Fig. 2), only one mutation significantly correlated with major PI/r resistance mutations. More precisely, Gag mutation P453L positively correlated with seven major protease resistance mutations (L33F, M46I, I54L, I54V, V82A, V82T, I84V). Several studies have shown a positive correlation of Gag P453L mutation with some protease major resistance mutations such as I50V and I84V^11,12,22^. The involvement of this mutation in PI/r resistance has been demonstrated in vitro and has been incriminated in contributing to the restoration of viral fitness^18^. The strong correlation of this mutation with PI resistance mutations has been confirmed by the dendrogram; where P453L Gag mutation clustered with two major protease resistance mutations (M46I and I84V) (Fig. 4). This mutation although classified as PI/r-associated mutation^21^ should be investigated to better understand its likely involvement in PI/r resistance. Among the mutations which did not correlate with any major or accessory protease resistance mutations, E460A was described to be repeatedly associated with therapy failure^23^, but its role in viral fitness and or drug resistance has not yet been proven. Gag mutations R464G, S465F, V467E, Q474P, I479R, and E482G were also previously identified as polymorphisms associated to PI/r exposure^21^. Thus, the potential role of these Gag mutations in the pathways to the development of resistance still need to be confirmed.

Furthermore, by comparing resistance mutation profile of RTI treated patients versus ART-naïve patients, we found the presence of certain Gag mutations (L449F, I479R, E482G and Y484P) significantly associated to RTI treated patients, and which were also significantly associated to PI/r treated patients. This suggest that some Gag mutations in patients failing PI/r may have been previously induced during RTI treatment, showing a possible interactive role of RTI on emerging Gag mutants. As previously reported by Soldi et al. in a subtype F highly-prevailing setting, the presence of NRTI mutations was associated to some PI/r resistance mutations (i.e. I50LV) in patients failing PI/r treatment^24^. Indeed, some studies revealed that some inserts at the P6 region within the Gag gene may favor virus escape from nucleoside reverse transcriptase inhibitors (NRTI) through greater accumulation of resistance mutations, leading to high level of resistance to this drugs class^25^. Of the 18 Gag mutations associated to PI/r exposure, fifteen were not significantly present among patients on RTI when compared to naïve patients, and ten mutations were significantly associated to PI/r exposure when compared PI/r-treated vs RTI-treated. This reinforces the fact that these mutations are primarily selected under PI/r exposure.

In this study, we observed that the distribution of certain Gag mutations was significantly different according to the HIV-1 non-B viral subtypes, which seem to harbor more than two substitutions in P2/NC Gag cleavage site compare to B subtype^26^. Data on the distribution of Gag mutations potentially associated with drug susceptibility among non-B subtypes are limited. Our analysis showed that subtypes A and G, although not in large numbers seem to be most affected (Table 2). The association of Gag mutations in class 1 (< 1% variability among drug-naïve individuals that significantly increased with PI treatment) among some of these non-B subtypes deserves to be further investigated. Of note, some differences in the susceptibility to PIs of certain non-B subtypes such CRF02_AG and G when compared to other subtypes was previously documented in the literature^27^.

Among Gag mutations which positively correlated with major protease drug resistance mutations, we did not find any significant association with a worse virologic or immunologic outcome. However, Gag mutations L449F and Y484P had higher median viremia when compared to individuals with a wild-type residues. Of note L449F were described to contribute to full recovery of viral fitness in protease inhibitor resistance^13,28^.

The significantly associated Gag gene mutations described to PI/r regimen in this non-B subtypes population are similar to those described in several studies conducted among B subtypes^28^. The selection of Gag gene mutations related to PI/r exposure would therefore be similar in B and non-B subtypes in this C-terminal Gag region. These prominent findings henceforth support the need for a cohort-study in order to monitor the emergence of these mutations over time, the dynamics of immuno-virological parameters, in order to generate clinical confirmations that would be underscored by advanced in vitro analyses (by phenotyping via viral culture). Because we compared the non-B subtypes altogether, without a paired-wise comparison to B subtype, this could shadow some insightful information in non-B that would have reinforced our study-findings.

## Conclusion

In summary, we have found that some Gag P7 (NC)-P6 mutations are associated with an increased frequency at PI/r failure among HIV-1 non-B isolates. In particular, mutations L449F, P453L and Y484P have shown a significant correlation with several major and/or accessory protease resistance mutations. The potential implication of novel Gag P7 (NC)-P6 mutations in treatment failure under PI/r-based regimen deserves to be further investigated in each non-B subtype and using cohort studies adding to in vitro experiments. These mutations could have clinical implications, since the current level of potential PI/r drug resistance might be underestimated.

## Materials and methods

### Specimen used for analysis

The study was performed on plasma samples of PLHIV failing their treatment. They were collected from January 2018 through December 2020 in Cameroon for routine clinical monitoring of HIV genotypic drug resistance at the Virology Laboratory of the Chantal BIYA International Reference Centre for research on the HIV/AIDS prevention and management (CIRCB) in Yaoundé-Cameroon. Patients were either naïve to antiretrovirals or treated with a PI/r-based regimen or a reverse transcriptase inhibitor (RTI) based regimen. Only patients with a clearly documented treatment history (available in their medical record) were enrolled; all participants on treatment were experiencing virological failure (i.e. a sustained plasma viral load > 1000 copies/ml after enhanced adherence counseling); and these participants on ART were considered to be adherent, based on self-reporting and enhanced adherence support according to national guidelines^29^.

### HIV sequencing

For the sequences obtained, HIV-1 P7 (NC)–P6 Gag and protease genotyping was performed as previously described by Teto et al.^30^. Briefly, after viral RNA extraction from plasma samples, RNA was reverse-transcribed and amplified. From positive amplicons, DNA sequencing was performed in both sense and antisense using eight overlapping sequence specific primers. Sequences were obtained after capillary electrophoresis on Applied Biosystem™ 3500 genetic analyzer (Applied Biosystems™, USA), and sequences of approximately 168 nucleotides of the Gag gene P7 (NC)-P6) and 297 nucleotides for protease region were assembled and manually edited using Seqscape v.2.6. for P7 (NC)-P6 Gag gene and RECall (CDC, Atlanta) for protease gene.

### Subtyping and drug resistance determination

Nucleotide sequences were aligned with subtype/CRFs reference sequences from the Los Alamos National Laboratory (LANL) database using the CLUSTAL.W integrated into Bioedit version 7.2.5 software (https://bioedit.software.informer.com/7.2/). Following comparison of each sequence to the subtypes and CRFs reference sequences (database accessed on 8/17/2021), gaps were removed from the final alignments. The phylogenetic tree was constructed by the neighbor-joining and Kimura’s two-parameter methods^31^ using the MEGA version 5.0 software (https://www.megasoftware.net/). The reliability of the branching orders was determined using 70% bootstrap robustness for subtype assignation.

#### Mutation’s prevalence

To assess the association of Gag mutations with PI/r exposure, we calculated their respective frequencies in isolates from 101 drug-naïve patients, 118 patients on RTI, and 143 patients on PI/r-based regimen. We then performed chi-squared tests of independence (based on a 2X2 contingency table) to verify statistically significant differences in frequency between the following groups of patients: (i) drug-naïve patients versus PI/r treated patients, (ii) drug-naıve patients versus patients on RTIs; (iii) patients on RTIs versus patients on PI/r.

Regarding protease mutations, we focused our attention on PI/r major and accessory drug resistance mutations according to the Stanford list of mutations (https://hivdb.stanford.edu/hivdb/by-mutations/). In our analysis, the Cochran rule, which is a conventional criterion for the chi-squared test to be valid, was fully satisfied. In fact, in each contingency table performed with our data set, 72% of the expected frequencies exceed 5, and all the expected frequencies exceed 1. In addition, in those few cases where the expected frequency in a single cell of the contingency table was less than 5, the significance was also confirmed by using the Monte Carlo significance test procedure^32^.

We used the Benjamini–Hochberg method^33^ to identify results that were statistically significant in the presence of multiple-hypothesis testing. A false discovery rate of 0.05 was used to determine statistical significance.

#### Mutation’s covariation

In the set of 143 PI/r treated patients, we exhaustively analyzed patterns of pairwise interactions among Gag mutations associated PI/r treatment and Protease mutations. Specifically, for each pair of mutations and corresponding wild-type residues, Fisher’s exact test was performed to assess whether co-occurrence of the mutated residues differed significantly from what would be expected under an independence assumption. Again, the Benjamini–Hochberg method was used to correct for multiple testing, here at a false discovery rate of 0.01. Samples having a mixture of two or more mutations at a given pair of positions were ignored in calculating the covariation, since it is not possible to identify whether these mutations are indeed located in the same viral genome.

#### Cluster analysis

To analyze the covariation structure of mutations in more detail, we performed average linkage hierarchical agglomerative clustering, as described elsewhere^34^. Hierarchical clustering methods, which under different names are also widely used in phylogenetic tree building, rely on a matrix of pairwise dissimilarities between entities. Briefly, in average linkage clustering, clusters of increasing size are formed starting from one-element groups by iteratively joining two clusters with minimum average inter cluster distances between pairs of mutations. The distance between a pair of mutations was derived from the phi correlation coefficient, which is a measure of the association between two binary random variables, with 1 and − 1 representing maximal positive and negative association, respectively. This similarity measure was transformed into a distance by mapping phi = 1 to distance 0 and phi = − 1 to distance 1, with linear interpolation in between. The distance between different mutations at a single position was left undefined, as such pairs never co-occur in a single sequence (except from mixtures) and would lead to distorted dendrograms owing to their great distance. sed on which groups are associated into hierarchical clusters of decreasingly strong association. To assess the stability of the resulting dendrogram, confidence values for all sub trees in the dendrogram were computed by 100 replications of the clustering procedure on sequence sets bootstrapped from the original 143 sequences^34^. For instance, a bootstrap value of 1 simply means that out of 100 runs, all 100 had these two mutations (or groups of mutations) most closely linked. In this dendrogram, only Gag gene mutations significantly associated with PI/r exposure and major/accessory protease resistance mutations were considered.

### Ethical approval and informed consent

This study was conducted in accordance with the Declaration of Helsinki. The study protocol was approved by the Cameroon National Ethics Committee, all subjects gave written informed consent/assent for inclusion before participating in the study.
